# *SLC12A3* Variation and Renal Function in Chinese Patients With Hypertension

**DOI:** 10.3389/fmed.2022.863275

**Published:** 2022-06-21

**Authors:** Chin-Chou Huang, Chia-Min Chung, Chih-Yu Yang, Hsin-Bang Leu, Po-Hsun Huang, Liang-Yu Lin, Tao-Cheng Wu, Shing-Jong Lin, Wen-Harn Pan, Jaw-Wen Chen

**Affiliations:** ^1^Division of Cardiology, Department of Medicine, Taipei Veterans General Hospital, Taipei, Taiwan; ^2^School of Medicine, National Yang Ming Chiao Tung University, Taipei, Taiwan; ^3^Institute of Pharmacology, School of Medicine, National Yang Ming Chiao Tung University, Taipei, Taiwan; ^4^Cardiovascular Research Center, National Yang Ming Chiao Tung University, Taipei, Taiwan; ^5^Center for Drug Abuse and Addiction, China Medical University Hospital, Taichung, Taiwan; ^6^Graduate Institute of Biomedical Sciences, China Medical University, Taichung, Taiwan; ^7^Institute of Clinical Medicine, National Yang Ming Chiao Tung University, Taipei, Taiwan; ^8^Division of Nephrology, Department of Medicine, Taipei Veterans General Hospital, Taipei, Taiwan; ^9^Healthcare and Services Center, Taipei Veterans General Hospital, Taipei, Taiwan; ^10^Department of Critical Care Medicine, Taipei Veterans General Hospital, Taipei, Taiwan; ^11^Division of Endocrinology and Metabolism, Department of Medicine, Taipei Veterans General Hospital, Taipei, Taiwan; ^12^Taipei Heart Institute, Taipei Medical University, Taipei, Taiwan; ^13^Institute of Biomedical Sciences, Academia Sinica, Taipei, Taiwan; ^14^Institute of Epidemiology, School of Public Health, National Taiwan University, Taipei, Taiwan

**Keywords:** glomerular filtration rate, glomerular hyperfiltration, hypertension, hypertensive nephropathy, SLC12A3

## Abstract

**Objective:**

*SLC12A3* (solute carrier family 12 member 3) gene variants are associated with diabetic nephropathy; however, their association with hypertensive nephropathy remains unknown. We aimed to investigate the association between *SLC12A3* gene polymorphisms and renal function in patients with hypertension.

**Methods:**

Participants from three non-diabetic hypertensive cohorts, including young-onset hypertension (cohort 1, *n* = 882), treatment-naïve hypertension (cohort 2, *n* = 90), and follow-up cohort (cohort 3, *n* = 166), underwent genotyping for single nucleotide polymorphisms in *SLC12A3*. Renal events were defined as a >25 and >50% decline in estimated glomerular filtration rate (eGFR).

**Results:**

In cohort 1, *SLC12A3* rs16963397 C/C or C/G (*P* = 0.005), rs13334864 C/C or C/T (*P* = 0.020), and rs7187932 A/A or A/G polymorphisms (*P* = 0.014) had higher eGFRs compared to their counterparts, with similar findings observed in cohort 2. In cohort 3, over a mean follow-up of 5.8 ± 1.7 years, participants with either *SLC12A3* rs16963397 C/C or rs13334864 C/C polymorphisms had more >25 and >50% eGFR decline than their counterparts (log-rank test, *P* = 0.058 and *P* = 0.038, respectively). Cox regression analysis revealed that *SLC12A3* rs16963397 C/C and rs13334864 C/C polymorphisms were significantly associated with an increased risk of >25% [hazard ratio (HR), 3.294; 95% confidence interval (CI), 1.158–9.368; *P* = 0.025] and >50% decline in eGFR (HR, 18.630; 95% CI, 1.529–227.005, *P* = 0.022) than their counterparts.

**Conclusion:**

*SLC12A3* polymorphisms are associated with renal function in Chinese patients with hypertension.

## Introduction

Hypertension is an important public health challenge worldwide (1, 2). More than one-fourth of the global adult population has hypertension, and its incidence is rising (3). Hypertension is one of the most important factors for chronic kidney disease (CKD) and end stage renal disease (ESRD), and the importance of hypertension management in renal protection cannot be overemphasized (4, 5).

The *SLC12A3* (solute carrier family 12 member 3) gene is located on chromosome 16 (16q13). *SLC12A3* encodes the thiazide-sensitive Na-Cl cotransporter (NCC) in the luminal membrane of the distal convoluted tubule (6, 7). Mutations in NCC have been reported to be responsible for Gitelman's syndrome (8, 9), an autosomal recessive renal tubular disorder characterized by hypokalemia, hypomagnesemia, hypocalciuria, and metabolic alkalosis. It has also been reported that *SLC12A3* polymorphism is linked to the effects of thiazide diuretics (10).

Several studies have reported a link between *SLC12A3* gene variants and diabetic nephropathy (11–18). However, the association between genetic variations in *SLC12A3* and hypertensive nephropathy remains unknown. Therefore, we aimed to investigate the role of *SLC12A3* gene polymorphisms on renal function in non-diabetic hypertensive patients in a Chinese population.

## Materials and Methods

### Study Population

**Figure 1:**
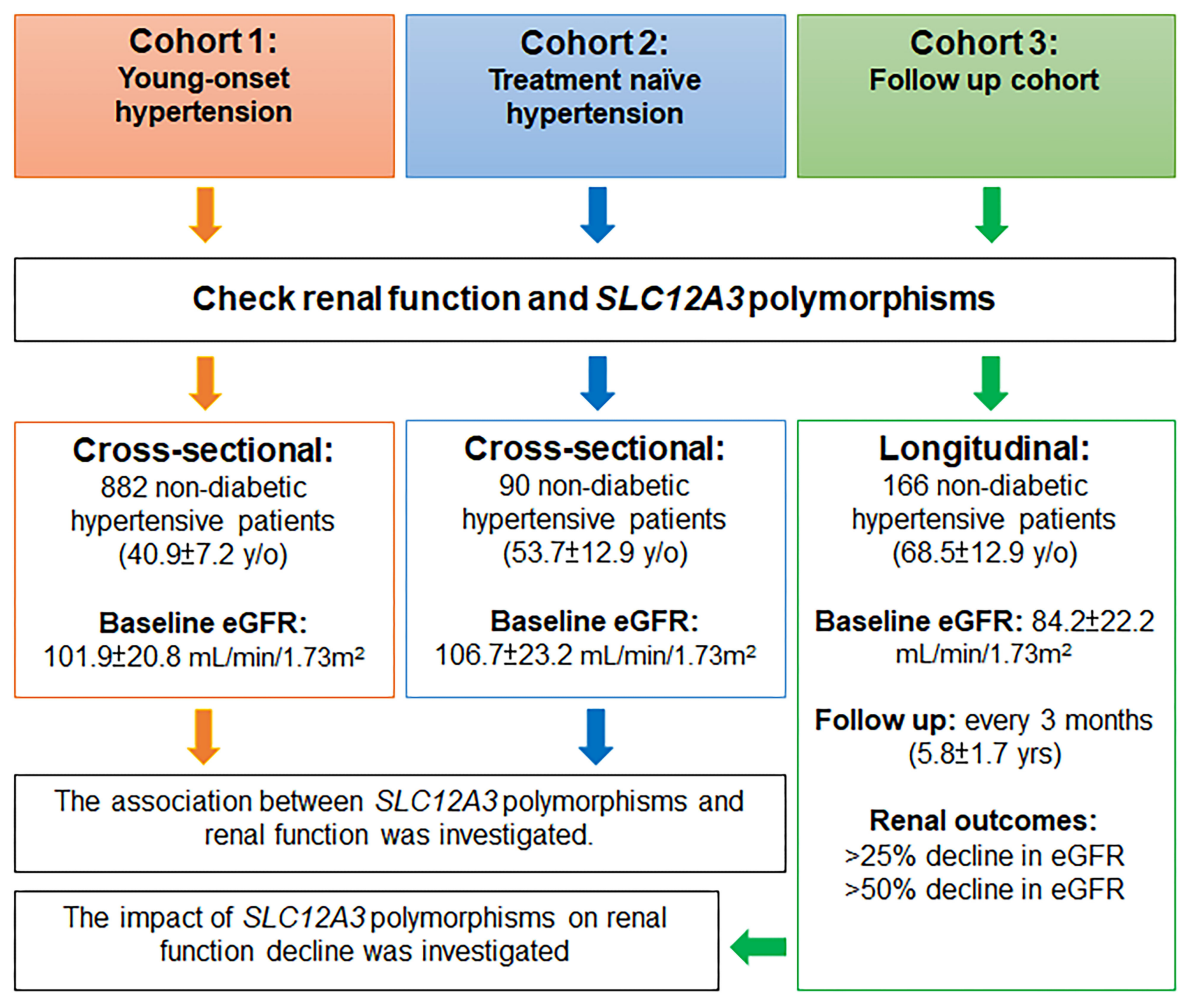
Flow chart of the study.

The study involved three non-diabetic hypertensive cohorts, including a young-onset hypertension (YOH) cohort (cohort 1), a treatment-naïve cohort (cohort 2), and a follow-up cohort (cohort 3) (Figure 1).

In cohort 1, non-diabetic participants with YOH were recruited from six medical centers in Taiwan between 2004 and 2005. The inclusion criteria were as follows: age 20–50 years; subjects who met one of the following criteria: (i) subject's systolic blood pressure (SBP) ≥ 140 mmHg or diastolic blood pressure (DBP) ≥90 mmHg in at least two consecutive visiting 2 months, (ii) subject taking one anti-hypertensive medication in 2 months; body mass index (BMI) ≤ 35 kg/m^2^; fasting glucose level < 126 mg/dL with no diabetes mellitus; no medical history of severe diseases; and no acute disease in the 2 weeks prior to the visit. Participants with secondary hypertension were excluded from the study (19).

In cohort 2, participants with newly diagnosed hypertension who were previously untreated were prospectively included in the study if all of the following criteria were fulfilled: age ≥ 25 years; a sitting office SBP of 140–180 mmHg and/or a DBP of 90–110 mmHg on three different occasions within 3 months before the study, fasting plasma sugar level <126 mg/dL with no diabetes mellitus, and no clinical evidence of secondary hypertension. Participants with the following characteristics were excluded: current use of antihypertensive drugs; history of major systemic disease in the 3 months prior to the study; renal dysfunction with a plasma creatinine level of >1.7 mg/dL; liver dysfunction with liver enzyme activity of more than double the normal upper limit; congestive heart failure with New York Heart Association function class II-IV; and pregnancy.

In cohort 3, participants diagnosed with hypertension from February 2012 to December 2020 at the Taipei Veteran General Hospital were included. The inclusion criteria were as follows: age ≥ 20 years; essential hypertensive patients; no medical history of severe diseases; and no acute disease in the 2 weeks prior to the study. Participants with secondary hypertension were excluded from the study (20). Only participants without diabetes mellitus were included in the present study.

The study protocols were approved by the Ethics Committee of the Academic Sinica and Taipei Veterans General Hospital. All participants agreed to participate after being informed of the nature and purpose of the study. This study was conducted in accordance with the principles of the Declaration of Helsinki.

### Study Design

A flow chart of the study is presented in Figure 1. This study was conducted using three non-diabetic hypertensive cohorts. In cohorts 1 and 2, two cross-sectional cohorts, the association between *SLC12A3* polymorphisms and renal function was investigated in participants with YOH (cohort 1) and treatment-naïve hypertension (cohort 2). In cohort 3, a longitudinal cohort, the potential impact of *SLC12A3* polymorphisms on renal function decline was investigated in participants with essential hypertension. Renal function was assessed every 3 months.

Prescribed antihypertensive agents were recorded, including angiotensin-converting enzyme inhibitors (ACEIs), angiotensin receptor blockers (ARBs), beta-blockers, calcium channel blockers (CCBs), and thiazide diuretics. All participants underwent genotyping for single nucleotide polymorphisms (SNPs) in the *SLC12A3* gene.

### Blood Pressure Measurements

Office BP was measured according to a standardized protocol by a well-trained nurse with an electronic BP monitor (cohort 1, Omega 1400 NBP, Invivo Research Inc., Orlando, FL, USA; cohorts 2 and 3, Omron HEM-7121, Omron Healthcare Taiwan Co., Songshan, Taipei, Taiwan, ROC) in the morning after participants had been instructed to sit for 10 min in a quiet room. Three consecutive BP measurements were obtained from the same upper arm, with each measurement taken at 30-s intervals.

For ambulatory BP monitoring (ABPM), participants were connected to an ABPM device between 08:00 and 10:00 h. The device was programmed to record BP every 15 min between 06:00 and 22:00 h (daytime BP), and every 30 min from 22:00 to 0600 h (nighttime BP).

### Laboratory Measurements

Blood samples were collected in the morning after overnight fasting. All blood samples were sent to the central laboratory for analysis. The participants were instructed to take all routine medications, as they normally would. The blood samples were centrifuged, and the serum was thawed for analysis. The estimated glomerular filtration rate (eGFR) was calculated using the four-variable equation proposed by the Modification of Diet in Renal Disease Study (21).

### Twenty-Four-Hour Urine Collection

In cohort 1, every participant was provided with a urine sample container. Urine samples were collected over a 24-h period by a well-trained nurse. Complete oral and written guidance about urine specimen collection, transportation, and preservation were also provided. On the day of urine collection, the participants were required to follow their daily diet habits, and they were advised to avoid strenuous exercise in order to reduce sweating. The participants were required to discard the first voided urine upon waking up in the morning and to collect all voided urine during the subsequent 24 h, including the first void sample of the following morning. Upon completion of collection, a well-trained nurse recorded the urine volume in each collection container to determine the total urine volume during the 24-h collection period. All urine samples were sent to a central laboratory to determine the levels of urinary sodium, potassium, chloride, and creatinine.

### Selection of Candidate Gene

The *SLC12A3* gene was evaluated in this study. We searched for SNPs in the gene using the NCBI SNP database (http://www.ncbi.nlm.nih.gov/SNP/) and selected SNPs with a minor allele frequency >0.05 as the genotyping markers. We investigated six tag SNP markers in the introns of the *SLC12A3* gene, including rs16963397, rs13334864, rs7187932, rs12449275, rs12447287, and rs1138429.

### Genotyping

A total of 20 cc of blood was collected from each participant. Genomic DNA was isolated from peripheral lymphocytes using the phenol/chloroform extraction method. SNP genotyping was performed using high-throughput matrix-assisted laser desorption and ionization–time of flight (MALDI-TOF) mass spectrometry. Briefly, primers and probes were designed using SpectroDESIGNER software (Sequenom, San Diego, California, USA). Multiplex PCRs were performed, and unincorporated ddNTPs were dephosphorylated using shrimp alkaline phosphatase (Hoffman-LaRoche, Basel, Switzerland), followed by primer extension. The purified primer extension reaction was spotted onto a 384-element silicon chip (Spectro-CHIP, Sequenom) and analyzed using an autoflex MALDI-TOF SpectroREADER mass spectrometer (Sequenom); the resulting spectra were processed with SpectroTYPER (Sequenom). The people who performed the genetic study were blinded to the clinical data of the study subjects.

### Renal Outcomes

The participants in cohort 3 were followed up to assess renal function decline. Renal events during the follow-up period were defined as minor nephropathy, >25% decline in eGFR, and major nephropathy, >50% decline in eGFR; these definitions have been used previously (20, 22).

### Statistical Analysis

The participant characteristics were summarized using descriptive statistics. Quantitative variables are expressed as the mean ± standard deviation (SD), and categorical variables are expressed as frequencies (percentages). Parametric continuous data between different groups were compared using Student's *t*-test. Non-parametric data between different groups were compared using the Mann–Whitney test. Categorical variables were analyzed using the chi-square test or Fisher's exact test.

In cohort 1, genetic association analyses were conducted using a general linear model to evaluate the relationship between eGFR and additive, dominant, and recessive model assumptions. Statistical significance was determined according to the lowest *P*-value (*P* < 0.05) in the three models. Multivariate analysis was performed using linear regression after adjusting for potential confounding factors, including age, sex, BMI, office BP ≥ 140/90 mmHg, ACEI/ARB, beta-blocker, CCB, thiazide diuretics, and baseline eGFR.

In cohort 3, survival analysis was assessed using the Kaplan–Meier curve, with significance based on the log-rank test. Cox proportional hazard regression analysis was performed to assess the independent effects of *SLC12A3* polymorphism and renal outcomes. The adjusted hazard ratios (HRs) with 95% confidence intervals (CIs) were estimated after adjusting for potential confounding factors, including age, sex, BMI, office BP ≥ 140/90 mmHg, ACEI/ARB, beta-blocker, CCB, thiazide diuretics, and baseline eGFR.

Statistical significance was defined as a two-sided *P* < 0.05. Statistical analysis was performed using SPSS software (Version 21.0, SPSS Inc., Chicago, IL, USA).

## Results

### *SLC12A3* Polymorphisms and Renal Function in Patients With YOH: Cohort 1

In cohort 1, 882 non-diabetic participants with YOH were genotyped for *SLC12A3*. The mean age of the participants was 40.9 ± 7.2 years, and ~69.0% were men. The average BMI of the participants was 26.5 ± 3.5 kg/m^2^, the mean SBP was 129.1 ± 14.6 mmHg, and the mean DBP was 86.2 ± 11.6 mmHg. The use of antihypertensive agents included ACEIs/ARBs (42.7%), beta-blockers (45.1%), CCBs (41.8%), and thiazide diuretics (16.2%). The serum creatinine level was 0.8 ± 0.2 mg/dL, and the eGFR was 101.9 ± 20.8 mL/min/1.73 m^2^ (Table 1, Figure 1).

**Table 1:** Baseline characteristics of the participants.

	**Cohort 1**	**Cohort 2**	**Cohort 3**
Patient number, *n*	882	90	166
Age, years	40.9 ± 7.2	53.7 ± 12.9	68.5 ± 12.9
Male, *n* (%)	609 (69.0%)	45 (50.0%)	99 (59.6%)
BMI, kg/m^2^	26.5 ± 3.5	25.8 ± 3.8	25.9 ± 3.8
Office SBP, mmHg	129.1 ± 14.6	143.6 ± 17.2	130.5 ± 17.3
Office DBP, mmHg	86.2 ± 11.6	93.0 ± 12.3	80.0 ± 11.1
ACEI/ARB, *n* (%)	377 (42.7%)	–	102 (61.4%)
Beta-blocker, *n* (%)	398 (45.1%)	–	44 (26.5%)
CCB, *n* (%)	369 (41.8%)	–	115 (69.3%)
Thiazide, *n* (%)	143 (16.2%)	–	57 (34.3%)
eGFR, mL/min/1.73 m^2^	101.9 ± 20.8	106.7 ± 23.2	84.2 ± 22.2
Creatinine, mg/dL	0.8 ± 0.2	0.7 ± 0.2	0.9 ± 0.2
Mean follow up duration, years	–	–	5.8 ± 1.7

*ACEI, angiotensin converting enzyme inhibitor; ARB, angiotensin receptor blocker; BMI, body mass index; CCB, calcium channel blocker; DBP, diastolic blood pressure; eGFR, estimated glomerular filtration rate; SBP, systolic blood pressure*.

The genetic information regarding the *SLC12A3* polymorphisms in cohort 1 is presented in Table 2. Of the six SNPs in *SLC12A3*, rs16963397 (*r* = 0.094, *P* = 0.005), rs13334864 (*r* = 0.078, *P* = 0.020), and rs7187932 (*r* = 0.083, *P* = 0.014) polymorphisms were associated with eGFR (Table 2).

**Table 2:** Association of individual SLC12A3 gene variants and estimated glomerular filtration rate (cohort 1).

**SNP**	**Position**	**Role**	**Genotype**	**Frequency**	**Allele**	**MAF**	**HWE**	***r***	***P*-value**
rs16963397	chr16:56927441	Intron	C/C	55 (6.2%)	G:C	0.244	0.636	0.094	0.005
			C/G	320 (36.3%)					
			G/G	507 (57.5%)					
rs13334864	chr16:56929841	Intron	C/C	131 (14.9%)	T:C	0.406	<0.001	0.078	0.020
			C/T	325 (36.8%)					
			T/T	426 (48.3%)					
rs7187932	chr16:56931704	Intron	A/A	63 (7.1%)	G:A	0.245	0.073	0.083	0.014
			A/G	307 (34.8%)					
			G/G	512 (58.0%)					
rs12449275	chr16:56937855	Intron	A/A	35 (4.0%)	G:A	0.124	<0.001	0.044	0.188
			A/G	148 (16.8%)					
			G/G	699 (79.3%)					
rs12447287	chr16:56937998	Intron	C/C	43 (4.9%)	T:C	0.145	<0.001	0.054	0.109
			C/T	170 (19.3%)					
			T/T	669 (75.9%)					
rs1138429	chr16:56942921	Intron	A/A	626 (71.0%)	A:T	0.162	0.091	0.025	0.461
			A/T	226 (25.6%)					
			T/T	30 (3.4%)					

*HWE, Hardy-Weinberg equilibrium; MAF, minor allele frequency; r, correlation coefficient; SNP, single nucleotide polymorphisms.*

*Alleles shown are major:minor*.

Overall, participants with *SLC12A3* rs16963397 C/C or C/G polymorphisms, rs13334864 C/C or C/T polymorphisms, and rs7187932 A/A or A/G polymorphisms had higher eGFR and higher 24-h urine sodium excretion than their counterparts (Supplementary Tables 1–6).

Multivariate linear regression revealed that *SLC12A3* rs16963397 C/C or C/G polymorphisms (β = 3.183, 95% CI = 0.547–5.818, *P* = 0.018), *SLC12A3* rs13334864 C/C or C/T polymorphisms (β = 2.784, 95% CI = 0.180–5.388, *P* = 0.036), and *SLC12A3* rs7187932 A/A or A/G polymorphisms (β = 2.778, 95% CI = 0.133–5.423, *P* = 0.040) were independently associated with eGFR (Table 3).

**Table 3:** Multivariate analysis for estimated glomerular filtration rate (cohort 1).

	**Model 1**			**Model 2**		
	**β**	**(95% CI)**	***P*-value**	**β**	**(95% CI)**	***P*-value**
rs16963397 (C/C or C/G vs. G/G)	3.071	(0.464–5.679)	0.021	3.183	(0.547–5.818)	0.018
rs13334864 (C/C or C/T vs. T/T)	2.701	(0.119–5.283)	0.040	2.784	(0.180–5.388)	0.036
rs7187932 (A/A or A/G vs. G/G)	2.649	(0.034–5.264)	0.047	2.778	(0.133–5.423)	0.040

*β, unstandardized coefficient; CI, confidence interval.*

*Model 1: Adjusted for age and male.*

*Model 2: Adjusted for age, male, BMI, office BP ≥ 140/90 mmHg, ACEI/ARB, beta-blocker, CCB, and thiazide*.

### *SLC12A3* Polymorphisms and Renal Function in Patients With Treatment-Naïve Hypertension: Cohort 2

Cohort 2 comprised 90 non-diabetic participants with treatment-naïve hypertension. The mean age of the participants was 53.7 ± 12.9 years, and ~50.0% were men. The average BMI of the participants was 25.8 ± 3.8 kg/m^2^, the mean SBP before treatment was 143.6 ± 17.2 mmHg, and the mean DBP before treatment was 93.0 ± 12.3 mmHg. The serum creatinine level was 0.7 ± 0.2 mg/dL and the eGFR was 106.9 ± 23.0 mL/min/1.73 m^2^ (Table 1, Figure 1).

Overall, participants with *SLC12A3* rs16963397 C/C or C/G polymorphisms, rs13334864 C/C or C/T polymorphisms, and rs7187932 A/A or A/G polymorphisms had lower serum creatinine and/or higher eGFR than their counterparts (Supplementary Tables 7–12).

### *SLC12A3* Polymorphisms and Renal Function Decline: Cohort 3

In cohort 3, 166 participants with non-diabetic hypertension were included. The mean age of the participants was 68.5 ± 12.9 years, and approximately 59.6% were men. The average BMI of the participants was 25.9 ± 3.8 kg/m^2^, the baseline SBP was 130.5 ± 17.3 mmHg, and the baseline DBP was 80.0 ± 11.1 mmHg. The antihypertensive agents used included ACEIs/ARBs (61.4%), beta-blockers (26.5%), CCBs (69.3%), and thiazide diuretics (34.3%). The renal function of the participants upon enrollment was generally not impaired (serum creatinine level of 0.9 ± 0.2 mg/dL and eGFR of 84.2 ± 22.2 mL/min/1.73 m^2^) (Table 1, Figure 1).

Overall, the baseline renal function, either eGFR or serum creatinine, was similar between participants with different *SLC12A3* polymorphisms (Supplementary Tables 13–18).

**Figure 2:**
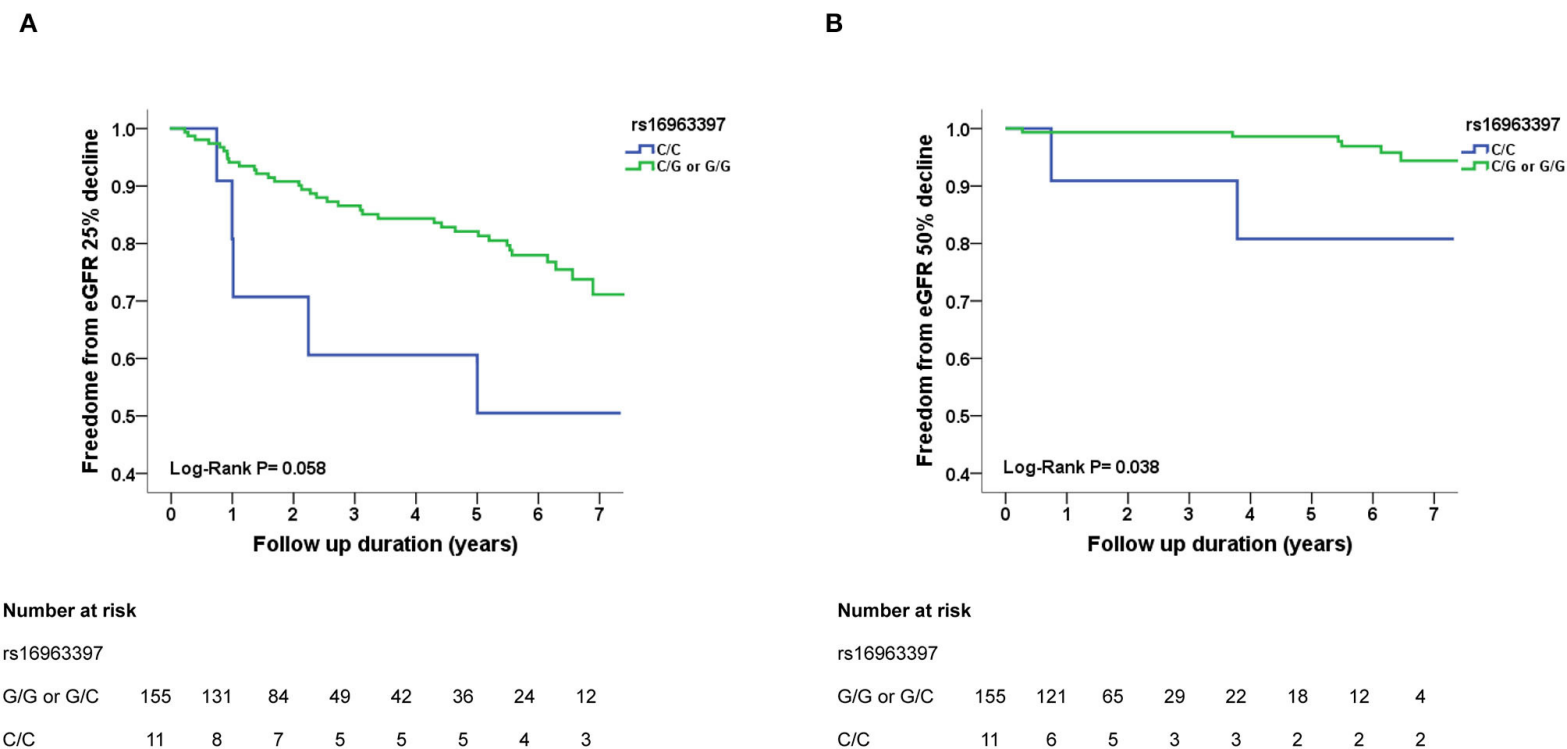
Kaplan–Meier survival curves showing the absence of renal events with respect to *SLC12A3* rs16963397 polymorphisms in patients with hypertension in cohort 3. All participants were divided into two groups according to *SLC12A3* rs16963397 polymorphisms. In panel A and B, the blue line represents the patient group with *SLC12A3* rs16963397 C/C polymorphisms, and the green line represents the group with *SLC12A3* rs16963397 C/G or G/G polymorphisms. Renal events were defined as >25% decline and >50% decline in estimated glomerular filtration rate (eGFR). Differences were compared using the log-rank test. **(A)** *SLC12A3* rs16963397 polymorphisms (C/C vs. C/G or G/G) and >25% decline in eGFR (*P* = 0.058). **(B)** *SLC12A3* rs16963397 polymorphisms (C/C vs. C/G or G/G) and >50% decline in eGFR (*P* = 0.038). The findings of *SLC12A3* rs16963397 C/C and *SLC12A3* rs13334864 C/C polymorphisms were the same.

During an average follow up period of 5.8 ± 1.7 years, >25% decline in eGFR was noted in 40 hypertensive participants and >50% decline in eGFR was noted in 8 hypertensive participants. Survival was assessed using Kaplan–Meier analysis. Participants with *SLC12A3* rs16963397 C/C polymorphism had more >25 and >50% eGFR decline than those with C/G or G/G polymorphisms, respectively (log-rank test, *P* = 0.058 and *P* = 0.038, respectively) (Figures 2A,B). Participants with the *SLC12A3* rs13334864 C/C polymorphism had more >25 and >50% eGFR decline than those with C/T or T/T polymorphisms (log-rank test, *P* = 0.058 and *P* = 0.038, respectively). The findings of *SLC12A3* rs16963397 C/C and *SLC12A3* rs13334864 C/C polymorphisms were the same. Participants with the *SLC12A3* rs7187932 A/A polymorphism had similar >25 and >50% eGFR decline to those with A/G or G/G polymorphisms (log-rank test, *P* = 0.340 and *P* = 0.298, respectively).

Cox regression analysis revealed that the *SLC12A3* rs16963397 C/C and rs13334864 C/C polymorphisms were significantly associated with an increased risk of >25% decline in eGFR (HR, 3.294; 95% CI, 1.158–9.368; *P* = 0.025) and >50% decline in eGFR (HR, 18.630; 95% CI, 1.529–227.005; *P* = 0.022) than their counterparts (Table 4). The findings of *SLC12A3* rs16963397 C/C and *SLC12A3* rs13334864 C/C polymorphisms were the same.

**Table 4:** *SLC12A3* gene polymorphisms and renal function decline (cohort 3).

	**eGFR** **>** **25% decline**		**eGFR** **>** **50% decline**	
	**HR (95% CI)**	***P*-value**	**HR (95% CI)**	***P*-value**
*SLC12A3* rs16963397 (C/C vs. C/G or G/G)	3.294 (1.158–9.368)	0.025	18.630 (1.529–227.005)	0.022
Age, years	1.037 (1.005–1.070)	0.021	1.114 (0.974–1.273)	0.115
Sex (male vs. female)	0.423 (0.211–0.847)	0.015	0.078 (0.008–0.797)	0.031
Body mass index, kg/m^2^	1.051 (0.967–1.142)	0.243	1.012 (0.810–1.264)	0.917
Office BP ≥ 140/90 mmHg	2.374 (1.210–4.655)	0.012	11.821 (1.558–89.687)	0.017
ACEI/ARB (yes vs. no)	0.814 (0.384–1.726)	0.591	0.244 (0.038–1.561)	0.136
Beta-Blocker (yes vs. no)	1.351 (0.665–2.746)	0.406	4.316 (0.734–25.376)	0.106
CCB (yes vs. no)	1.632 (0.709–3.756)	0.250	0.312 (0.038–2.554)	0.277
Thiazide (yes vs. no)	0.849 (0.387–1.861)	0.682	10.640 (1.535–73.734)	0.017
eGFR, mL/min/1.73 m^2^	1.041 (1.025–1.057)	<0.001	1.045 (1.001–1.092)	0.045
*SLC12A3* rs13334864 (C/C vs. C/T or T/T)	3.294 (1.158–9.368)	0.025	18.630 (1.529–227.005)	0.022
Age, years	1.037 (1.005–1.070)	0.021	1.114 (0.974–1.273)	0.115
Sex (male vs. female)	0.423 (0.211–0.847)	0.015	0.078 (0.008–0.797)	0.031
Body mass index, kg/m^2^	1.051 (0.967–1.142)	0.243	1.012 (0.810–1.264)	0.917
Office BP ≥140/90 mmHg	2.374 (1.210–4.655)	0.012	11.821 (1.558–89.687)	0.017
ACEI/ARB (yes vs. no)	0.814 (0.384–1.726)	0.591	0.244 (0.038–1.561)	0.136
Beta-blocker (yes vs. no)	1.351 (0.665–2.746)	0.406	4.316 (0.734–25.376)	0.106
CCB (yes vs. no)	1.632 (0.709–3.756)	0.250	0.312 (0.038–2.554)	0.277
Thiazide (yes vs. no)	0.849 (0.387–1.861)	0.682	10.640 (1.535–73.734)	0.017
eGFR, mL/min/1.73 m^2^	1.041 (1.025–1.057)	<0.001	1.045 (1.001–1.092)	0.045
*SLC12A3* rs7187932 (G/G vs. A/A or A/G)	2.408 (0.679–8.539)	0.174	6.103 (0.499–74.585)	0.157
Age, years	1.038 (1.006–1.070)	0.019	1.084 (0.967–1.215)	0.168
Sex (male vs. female)	0.436 (0.218–0.872)	0.019	0.146 (0.021–1.023)	0.053
Body mass index, kg/m^2^	1.041 (0.958–1.131)	0.348	0.978 (0.791–1.208)	0.835
Office BP ≥140/90 mmHg	2.314 (1.188–4.509)	0.014	8.275 (1.266–54.083)	0.027
ACEI/ARB (yes vs. no)	0.867 (0.408–1.843)	0.710	0.380 (0.066–2.192)	0.279
Beta-blocker (yes vs. no)	1.294 (0.638–2.622)	0.475	2.906 (0.561–15.067)	0.204
CCB (yes vs. no)	1.719 (0.748–3.948)	0.202	0.519 (0.079–3.426)	0.496
Thiazide (yes vs. no)	0.889 (0.407–1.942)	0.768	7.634 (1.301–44.806)	0.024
eGFR, mL/min/1.73 m^2^	1.041 (1.025–1.057)	<0.001	1.037 (0.997–1.080)	0.071

*ACEI, angiotensin-converting enzyme inhibitor; ARB, angiotensin receptor blocker; CCB, calcium channel blocker; CI, confidence interval; eGFR, estimated glomerular filtration rate; HR, hazard ratio; BP, blood pressure*.

## Discussion

This study aimed to examine the association between *SLC12A3* polymorphisms and renal function in Chinese patients with hypertension. To decrease the potential impact of diabetes mellitus, a well-known factor of renal dysfunction, this study was only conducted in non-diabetic participants. Furthermore, the association was tested in three cohorts, including a YOH cohort (cohort 1), a treatment-naïve cohort (cohort 2), and a follow-up cohort (cohort 3). *SLC12A3* rs16963397, rs13334864, and rs7187932 polymorphisms were found to be associated with eGFR in YOH and treatment-naïve hypertension. Furthermore, *SLC12A3* rs16963397 and rs13334864 polymorphisms were associated with renal function decline in participants with hypertension. As this is the first study to report the association between *SLC12A3* polymorphisms and renal function in patients with hypertension, further studies are still needed to further confirm our findings.

Although there have been some genetic studies of hypertensive nephropathy, these studies have mainly focused on the apolipoprotein L1 (*APOL1*) gene (23). Lipkowitz et al. reported that *APOL1* risk variants were significantly associated with CKD and kidney disease progression in African-American participants of the African American Study of Kidney Disease and Hypertension (AASK) trial (23). Parsa et al. further examined the effects of *APOL1* risk variants on CKD progression in the AASK trial and Chronic Renal Insufficiency (CRIC) study. The study found that *APOL1* risk variants were associated with higher rates of ESRD and progression of CKD in black patients compared to that in white patients (24). These findings suggest that genes other than *APOL1* might be related to hypertensive nephropathy in other non-African American populations.

Several studies have reported a link between *SLC12A3* gene variants and diabetic nephropathy (11–18). In a case-control study conducted in Japanese patients with diabetes, Tanaka et al. (11) reported that substitution of Arg913 with Gln in the *SLC12A3* gene might reduce the risk of developing diabetic nephropathy. In another case-control study conducted in Korean patients with diabetes, Kim et al. (12) reported that the *SLC12A3*-Arg913Gln variation was associated with ESRD caused by diabetic nephropathy. Moreover, in a case-control study conducted in a Malaysian population, Abu Seman et al. (13) reported that the *SLC12A3*-Arg913Gln variation was associated with diabetic nephropathy, and that the minor 913Gln allele of *SLC12A3* confers a protective effect in diabetic nephropathy. The roles of *SLC12A3* in kidney development and progression of diabetic nephropathy were further supported by animal studies in db/db mice and zebrafish. Similarly, two case-control studies also reported that *SLC12A3*-Arg913Gln variation was significantly associated with diabetic nephropathy in the Indian population (14, 15). Furthermore, a previous case-control study (16) also reported that the *SLC12A3*-Arg913Gln variation could be used to predict the risk of ESRD in Chinese patients with type 2 diabetes. In a 10-year longitudinal study of Japanese patients with type 2 diabetes, Nishiyama et al. (17) reported that the *SLC12A3*-Arg913Gln variation was linked with albumin excretion, and that the +78A allele may be protective against the development of diabetic nephropathy. However, in a case-control study conducted in Caucasians with type 2 diabetes, Ng et al. (18) found no association between *SLC12A3* gene variants and advanced diabetic nephropathy. Although the *SLC12A3* gene was found to be associated with diabetic nephropathy in some studies, the findings were not consistent with those of other studies. It is possible that the roles of *SLC12A3* in the progression of diabetic nephropathy are influenced by genetic differences.

Our study is the first to investigate the link between *SLC12A3* genetic variations and renal function decline in patients with hypertension. In the participants with YOH (cohort 1) and treatment-naïve hypertension (cohort 2), *SLC12A3* rs16963397 C/C or C/G polymorphisms, *SLC12A3* rs13334864 C/C or C/T polymorphisms, and *SLC12A3* rs7187932 A/A or A/G polymorphisms were associated with higher eGFR than their counterparts. In the follow-up cohort (cohort 3), participants with *SLC12A3* rs16963397 C/C and rs13334864 C/C polymorphisms were prone to develop renal function decline during an average follow-up period of 5.8 ± 1.7 years.

These findings might be explained by the hypothesis of glomerular hyperfiltration in hypertension (25–27). In cohort 1, the participants (40.9 ± 7.2 years old) were the youngest and the mean eGFR was 101.9 ± 20.8 mL/min/1.73 m^2^. The eGFR in males (99.0 ± 19.3 mL/min/1.73 m^2^) was at the high end of the anticipated eGFR; similarly, eGFR in females (108.4 ± 22.7 mL/min/1.73 m^2^) was higher than anticipated (25). In cohort 2, the participants (53.7 ± 12.9 years old) were older than those in cohort 1, and had higher eGFR than anticipated (all: 106.7 ± 23.2 mL/min/1.73 m^2^; male: 103.7 ± 17.7 mL/min/1.73 m^2^; female: 109.8 ± 27.6 mL/min/1.73 m^2^) (25). The participants in cohort 3 were the oldest (68.5 ± 12.9 years old); although the initial eGFR of the participants was within the range of the anticipated eGFR (all: 84.2 ± 22.2 mL/min/1.73 m^2^; male: 86.0 ± 23.0 mL/min/1.73 m^2^; female: 86.0 ± 24.0 mL/min/1.73 m^2^) (25), participants with *SLC12A3* rs16963397 C/C and *SLC12A3* rs13334864 C/C polymorphisms had faster eGFR reduction than their counterparts. These findings suggested that *SLC12A3* polymorphisms were associated with more significant glomerular hyperfiltration in the earlier stage of hypertension (cohorts 1 and 2), and resulted in a more rapid decline in eGFR in the later stage of hypertension (cohort 3) (Supplementary Figures 1, 2).

Furthermore, in cohort 1, we found that participants with *SLC12A3* rs16963397 C/C or C/G polymorphisms, *SLC12A3* rs13334864 C/C or C/T polymorphisms, and *SLC12A3* rs7187932 A/A or A/G polymorphisms had higher 24-h sodium excretion than their counterparts. Previous studies have reported that high urine sodium excretion is associated with a faster decline in renal function (28). Therefore, these findings suggest that these *SLC12A3* polymorphisms associated with higher 24-h sodium excretion may result in a faster decline in renal function. However, increased urine sodium excretion is associated with increased sodium intake (29, 30). Further studies are needed to clarify the relationship between *SLC12A3* polymorphisms, sodium intake and excretion, and renal function decline in patients with hypertension.

### Study Limitations

The current study has several limitations that need to be considered. First, the sample size was relatively small. Although the *SLC12A3* rs16963397 C/C and rs13334864 C/C polymorphisms were significantly associated with an increased risk of >25% decline in eGFR (cohort 3), it might only indicate association but not cause-effect relationship and the finding might be under power. Further studies with larger sample sizes are warranted in the future, such as using the data from Taiwan biobank. Second, the renal function in the two cross-sectional cohorts (cohorts 1 and 2) was grossly intact. Further case-control studies comparing participants with intact renal function and established hypertension-related CKD are warranted. Third, it is impossible to exclude all other possible causes of renal function deterioration in addition to hypertension. Therefore, this study was conducted only in non-diabetic participants. Furthermore, the first part of the study was conducted in participants with YOH (cohort 1) to minimize possible comorbidities. Fourth, renal function could also be confounded by the use of antihypertensive drugs. Therefore, the second part of the study was conducted in participants with treatment-naïve hypertension (cohort 2) to eliminate the impact of antihypertensive drugs. Fifth, only six SNP markers in the introns of *SLC12A3* were selected in the present study, and more comprehensive genetic studies should be conducted in the future. Sixth, renal events were defined as >25 and >g50% decline in eGFR in the third part of the study (cohort 3). The variation in the definition of renal function impairment across studies may limit the usefulness of comparing results between studies. Seventh, many non-genetic factors might be involved in the progression of CKD (cohort 3). However, we had tried our best to enroll only non-diabetic patients with essential hypertension and no medical history of severe diseases. We had included age, sex, BMI, office BP, and antihypertensive drugs in the multivariate analysis. Finally, our hypertensive patients were limited to Chinese patients with hypertension in Taiwan. Given that the roles of *SLC12A3* may vary among different ethnicities, further studies in other ethnic cohorts are warranted.

## Conclusion

Genetic variations in *SLC12A3* are associated with renal function in Chinese hypertensive patients. Future investigations are mandated to elucidate the detailed pathway and identify potential therapeutic targets for prevention of renal function decline in patients with hypertension.

## Data Availability Statement

The original contributions presented in the study are included in the article/Supplementary Material, further inquiries can be directed to the corresponding author.

## Ethics Statement

The studies involving human participants were reviewed and approved by the Ethics Committee of the Academic Sinica and Taipei Veterans General Hospital. The patients/participants provided their written informed consent to participate in this study.

## Author Contributions

C-CH contributed to the conception and design of the study, data acquisition, analysis, and interpretation, and drafted and critically revised the manuscript. C-MC and C-YY contributed to the conception and design of the study, the interpretation of data, and drafted the manuscript. H-BL, P-HH, L-YL, T-CW, S-JL, W-HP, and J-WC contributed to data acquisition and drafted the manuscript. All authors gave final approval and agreed to be accountable for all aspects of work ensuring integrity and accuracy. All authors contributed to the article and approved the submitted version.

## Funding

This work was supported by research grants V101B-004, V102B-024, V103C-019, V104C-025, V106C-120, V108C-151, VGHUST108-G1-3-2, VTA108-V1-7-2, V110C-058, V111C-086, V111D63-002-MY2-1, and 111EA-014 from Taipei Veterans General Hospital, Taipei, Taiwan, and by research grants MOST106-2314-B-075-040 and MOST108-2314-B-075-062-MY3 from the Ministry of Science and Technology, Taiwan.

## Conflict of Interest

The authors declare that the research was conducted in the absence of any commercial or financial relationships that could be construed as a potential conflict of interest.

## Publisher's Note

All claims expressed in this article are solely those of the authors and do not necessarily represent those of their affiliated organizations, or those of the publisher, the editors and the reviewers. Any product that may be evaluated in this article, or claim that may be made by its manufacturer, is not guaranteed or endorsed by the publisher.
