# Supplementary material for: SLC12A3 Variation and Renal Function in Chinese Patients With Hypertension
Source: Front Med (Lausanne). 2022 Jun 21;9:863275. doi: 10.3389/fmed.2022.863275 (PMC9253539; doi:10.3389/fmed.2022.863275)
Supplement: Supplementary file 1 [file Data_Sheet_1.docx]

Supplementary Material

# Supplementary Figure


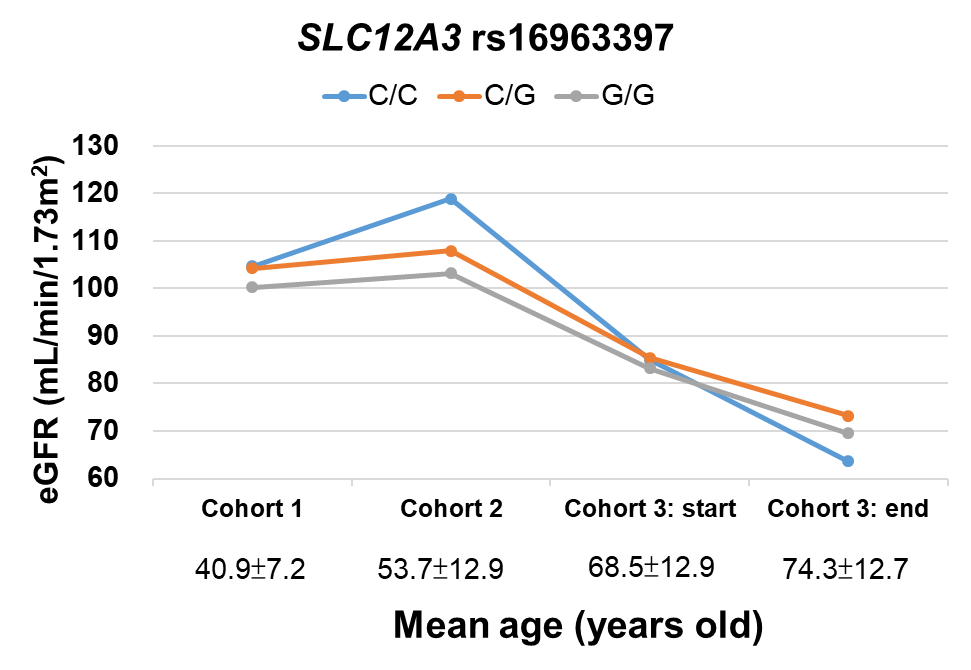


**Supplementary Figure 1.** Relationship between age and renal function with respect to *SLC12A3* s16963397 polymorphisms in patients with hypertension in the three cohorts. In cohort 1, significant differences in estimated glomerular filtration rate (eGFR) were observed in patients with *SLC12A3* rs16963397 C/C, C/G and G/G polymorphisms (C/C vs. C/G vs. G/G = 104.6 ± 19.9 vs. 104.2 ± 21.8 vs. 100.2 ± 20.1 mL/min/1.73 m^2^, *P* = 0.019). In cohort 2, participants with *SLC12A3* rs16963397 C/C had higher eGFR than those with C/G or G/G polymorphisms, although the difference was not significant (C/C vs. C/G vs. G/G = 118.8 ± 27.6 vs. 107.9 ± 26.1 vs. 103.3 ± 19.4 mL/min/1.73 m^2^, *P* = 0.149). In cohort 3, participants with *SLC12A3* rs16963397 C/C polymorphism had more rapid eGFR decline than those with C/G or G/G polymorphisms*.*


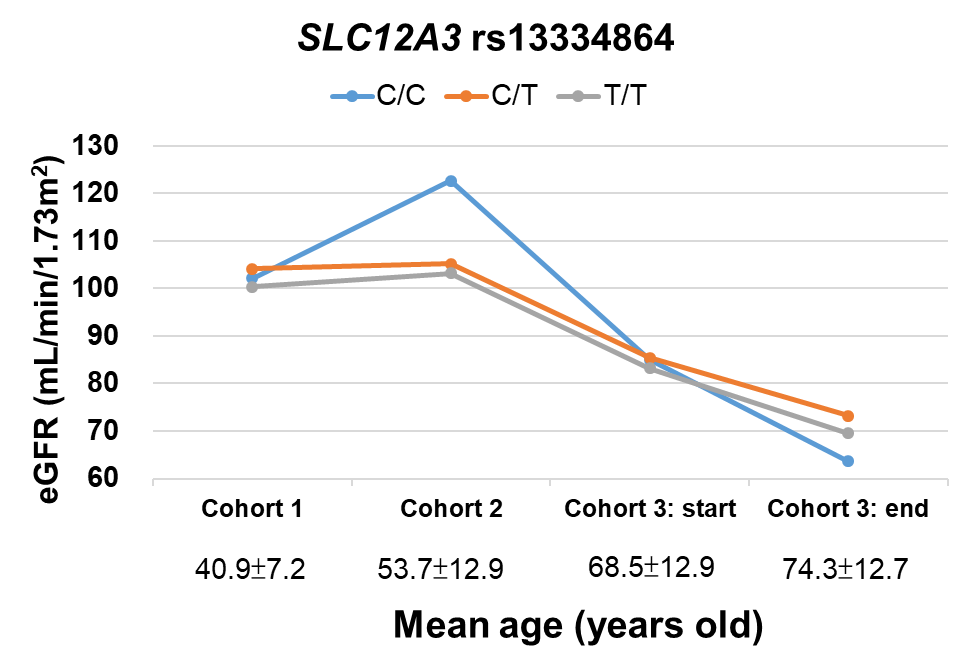


**Supplementary Figure 2.** Relationship between age and renal function with respect to *SLC12A3* rs13334864 polymorphisms in patients with hypertension in the three cohorts. In cohort 1, significant differences in estimated glomerular filtration rate (eGFR) were observed in *SLC12A3* rs13334864 C/C, C/T and T/T polymorphisms (C/C vs. C/T vs. T/T = 102.1 ± 20.8 vs. 104.1 ± 21.8 vs. 100.3 ± 20.0 mL/min/1.73 m^2^, *P* = 0.045). In cohort 2, significant differences in eGFR were observed (C/C vs. C/T vs. T/T = 122.6 ± 30.6 vs. 105.2 ± 23.0 vs. 103.2 ± 19.4 mL/min/1.73 m^2^, *P* = 0.025). In cohort 3, participants with *SLC12A3* rs13334864 C/C polymorphism had more rapid eGFR decline than those with C/T or T/T polymorphisms.

# Supplementary Tables

**Supplementary Table 1.** Baseline characteristics according to *SLC12A3* rs16963397 genotype (cohort 1)

| **rs16963397** | **C/C (n=55)** | **C/G (n=320)** | **G/G (n=507)** | ***P*-value** |
| --- | --- | --- | --- | --- |
| Age, years | 39.6±6.9 | 40.4±7.8 | 41.4±6.8 | 0.059 |
| Male, n(%) | 40 (72.7%) | 219 (68.4%) | 350 (69.0%) | 0.817 |
| BMI, kg/m^2^ | 26.2±3.3 | 26.4±3.6 | 26.5±3.4 | 0.787 |
| Office SBP, mmHg | 130.9±13.2 | 129.4±14.3 | 128.6±15.0 | 0.480 |
| Office DBP mmHg | 88.6±9.5 | 85.8±11.5 | 86.2±11.8 | 0.258 |
| 24-hour SBP, mmHg | 129.2±16.1 | 124.4±14.4 | 126.8±14.4 | 0.017 |
| 24-hour DBP, mmHg | 86.5±12.3 | 83.6±11.5 | 85.5±11.9 | 0.054 |
| Awake SBP, mmHg | 129.0±10.9 | 124.8±12.3 | 126.4±13.1 | 0.040 |
| Awake DBP, mmHg | 86.6±9.1 | 84.0±9.7 | 85.3±10.2 | 0.086 |
| Sleep SBP, mmHg | 116.8±11.2 | 113.2±12.4 | 114.2±13.1 | 0.125 |
| Sleep DBP, mmHg | 76.2±9.3 | 73.7±9.7 | 75.2±10.2 | 0.065 |
| ACEI/ARB, n(%) | 22 (40.0%) | 128 (40.0%) | 227 (44.8%) | 0.367 |
| Beta-blocker, n(%) | 27 (49.1%) | 122 (38.1%) | 249 (49.1%) | 0.007 |
| CCB, n(%) | 22 (40.0%) | 133 (41.6%) | 214 (42.2%) | 0.944 |
| Thiazide, n(%) | 10 (18.2%) | 48 (15.0%) | 85 (16.8%) | 0.734 |
| eGFR, mL/min/1.73m^2^ | 104.6±19.9 | 104.2±21.8 | 100.2±20.1 | 0.019 |
| Creatinine, mg/dL | 0.8±0.2 | 0.8±0.2 | 0.9±0.2 | 0.180 |
| Sodium, mmol/L | 141.5±2.7 | 141.1±2.9 | 141.3±2.5 | 0.576 |
| Potassium, mmol/L | 4.1±0.3 | 4.0±0.3 | 4.1±0.3 | 0.986 |
| Chloride, mmol/L | 102.5±2.6 | 102.0±2.9 | 102.2±2.5 | 0.456 |
| Uric acid, mg/dL | 6.6±1.7 | 6.7±1.7 | 6.6±1.7 | 0.676 |
| Total urine volume, mL | 1959.1±881.0 | 1914.8±724.5 | 1897.7±765.2 | 0.832 |
| Urine creatinine, mg/day | 1653.7±569.6 | 1514.2±487.1 | 1561.8±497.5 | 0.116 |
| Urine sodium, mmol/day | 175.2±67.5 | 174.3±68.6 | 164.7±72.9 | 0.130 |
| Urine potassium, mmol/day | 47.4±14.8 | 46.0±16.7 | 46.1±17.9 | 0.851 |
| Urine chloride, mmol/day | 156.9±59.8 | 153.9±61.6 | 147.0±68.2 | 0.240 |

ACEI, angiotensin converting enzyme inhibitor; ARB, angiotensin receptor blocker; BMI, body mass index; CCB, calcium channel blocker; DBP, diastolic blood pressure; eGFR, estimated glomerular filtration rate; SBP, systolic blood pressure.

**Supplementary Table 2.** Baseline characteristics according to *SLC12A3* rs16963397 genotype (cohort 1)

| **rs16963397** | **C/C+C/G (n=375)** | **G/G (n=507)** | ***P*-value** |
| --- | --- | --- | --- |
| Age, years | 40.3±7.7 | 41.4±6.8 | 0.025 |
| Male, n(%) | 116 (30.9%) | 157 (31.0%) | 0.992 |
| BMI, kg/m^2^ | 26.4±3.5 | 26.5±3.4 | 0.593 |
| Office SBP, mmHg | 129.6±14.1 | 128.6±15.0 | 0.321 |
| Office DBP mmHg | 86.2±11.3 | 86.2±11.8 | 0.957 |
| 24-hour SBP, mmHg | 125.1±14.7 | 126.8±14.4 | 0.086 |
| 24-hour DBP, mmHg | 84.1±11.6 | 85.5±11.9 | 0.078 |
| Awake SBP, mmHg | 125.4±12.2 | 126.4±13.1 | 0.252 |
| Awake DBP, mmHg | 84.4±9.7 | 85.3±10.2 | 0.193 |
| Sleep SBP, mmHg | 113.7±12.3 | 114.2±13.1 | 0.558 |
| Sleep DBP, mmHg | 74.1±9.7 | 75.2±10.2 | 0.105 |
| ACEI/ARB, n(%) | 150 (40.0%) | 227 (44.8%) | 0.157 |
| Beta-blocker, n(%) | 149 (39.7%) | 249 (49.1%) | 0.006 |
| CCB, n(%) | 155 (41.3%) | 214 (42.2%) | 0.794 |
| Thiazide, n(%) | 58 (15.5%) | 85 (16.8%) | 0.605 |
| eGFR, mL/min/1.73m^2^ | 104.2±21.6 | 100.2±20.1 | 0.005 |
| Creatinine, mg/dL | 0.8±0.2 | 0.9±0.2 | 0.067 |
| Sodium, mmol/L | 141.2±2.9 | 141.3±2.5 | 0.507 |
| Potassium, mmol/L | 4.1±0.3 | 4.1±0.3 | 0.987 |
| Chloride, mmol/L | 102.1±2.9 | 102.2±2.5 | 0.571 |
| Uric acid, mg/dL | 6.7±1.7 | 6.6±1.7 | 0.481 |
| Total urine volume, mL | 1921.3±748.4 | 1897.7±765.2 | 0.647 |
| Urine creatinine, mg/day | 1534.7±501.6 | 1561.8±497.5 | 0.426 |
| Urine sodium, mmol/day | 174.5±68.3 | 164.7±72.9 | 0.042 |
| Urine potassium, mmol/day | 46.2±16.4 | 46.1±17.9 | 0.975 |
| Urine chloride, mmol/day | 154.4±61.3 | 147.0±68.2 | 0.091 |

ACEI, angiotensin converting enzyme inhibitor; ARB, angiotensin receptor blocker; BMI, body mass index; CCB, calcium channel blocker; DBP, diastolic blood pressure; eGFR, estimated glomerular filtration rate; SBP, systolic blood pressure.

**Supplementary Table 3.** Baseline characteristics according to *SLC12A3* rs13334864 genotype (cohort 1)

| **rs13334864** | **C/C (n=131)** | **C/T (n=325)** | **T/T (n=426)** | ***P*-value** |
| --- | --- | --- | --- | --- |
| Age, years | 40.3±7.0 | 40.5±7.5 | 41.5±7.0 | 0.071 |
| Male, n(%) | 99 (75.6%) | 223 (68.6%) | 287 (67.4%) | 0.202 |
| BMI, kg/m^2^ | 26.4±3.5 | 26.4±3.6 | 26.5±3.4 | 0.919 |
| Office SBP, mmHg | 130.2±15.3 | 129.3±14.1 | 128.5±14.8 | 0.452 |
| Office DBP mmHg | 86.8±11.2 | 85.8±11.5 | 86.4±11.9 | 0.625 |
| 24-hour SBP, mmHg | 126.6±14.7 | 125.0±14.4 | 126.8±14.6 | 0.224 |
| 24-hour DBP, mmHg | 85.7±11.7 | 84.0±11.6 | 85.2±12.0 | 0.271 |
| Awake SBP, mmHg | 126.2±12.2 | 125.2±12.2 | 126.5±13.3 | 0.389 |
| Awake DBP, mmHg | 85.0±9.4 | 84.4±9.7 | 85.2±10.4 | 0.476 |
| Sleep SBP, mmHg | 114.3±12.6 | 113.8±12.8 | 114.1±12.9 | 0.900 |
| Sleep DBP, mmHg | 74.6±9.1 | 74.1±9.9 | 75.2±10.3 | 0.347 |
| ACEI/ARB, n(%) | 57 (43.5%) | 130 (40.0%) | 190 (44.6%) | 0.442 |
| Beta-blocker, n(%) | 65 (49.6%) | 123 (37.8%) | 210 (49.3%) | 0.004 |
| CCB, n(%) | 55 (42.0%) | 131 (40.3%) | 183 (43.0%) | 0.766 |
| Thiazide, n(%) | 26 (19.8%) | 47 (14.5%) | 70 (16.4%) | 0.364 |
| eGFR, mL/min/1.73m^2^ | 102.1±20.8 | 104.1±21.8 | 100.3±20.0 | 0.045 |
| Creatinine, mg/dL | 0.9±0.2 | 0.8±0.2 | 0.9±0.2 | 0.188 |
| Sodium, mmol/L | 141.4±2.2 | 141.1±3.0 | 141.3±2.5 | 0.521 |
| Potassium, mmol/L | 4.0±0.3 | 4.0±0.3 | 4.1±0.3 | 0.764 |
| Chloride, mmol/L | 102.4±2.2 | 102.1±3.0 | 102.1±2.5 | 0.415 |
| Uric acid, mg/dL | 6.7±1.6 | 6.7±1.7 | 6.6±1.8 | 0.744 |
| Total urine volume, mL | 1931.9±827.2 | 1928.2±758.4 | 1884.7±735.8 | 0.684 |
| Urine creatinine, mg/day | 1659.0±504.9 | 1515.4±485.7 | 1543.5±504.0 | 0.019 |
| Urine sodium, mmol/day | 172.0±66.1 | 175.2±68.9 | 163.0±73.9 | 0.059 |
| Urine potassium, mmol/day | 46.2±18.5 | 46.0±16.8 | 46.2±17.3 | 0.989 |
| Urine chloride, mmol/day | 153.0±58.7 | 154.8±62.0 | 145.7±69.6 | 0.146 |

ACEI, angiotensin converting enzyme inhibitor; ARB, angiotensin receptor blocker; BMI, body mass index; CCB, calcium channel blocker; DBP, diastolic blood pressure; eGFR, estimated glomerular filtration rate; SBP, systolic blood pressure.

**Supplementary Table 4.** Baseline characteristics according to *SLC12A3* rs13334864 genotype (cohort 1)

| **rs13334864** | **C/C+C/T (n=456)** | **T/T (n=426)** | ***P*-value** |
| --- | --- | --- | --- |
| Age, years | 40.4±7.4 | 41.5±7.0 | 0.022 |
| Male, n(%) | 134 (29.4%) | 139 (32.6%) | 0.298 |
| BMI, kg/m^2^ | 26.4±3.5 | 26.5±3.4 | 0.688 |
| Office SBP, mmHg | 129.6±14.5 | 128.5±14.8 | 0.268 |
| Office DBP mmHg | 86.1±11.4 | 86.4±11.9 | 0.688 |
| 24-hour SBP, mmHg | 125.5±14.5 | 126.8±14.6 | 0.179 |
| 24-hour DBP, mmHg | 84.5±11.6 | 85.2±12.0 | 0.367 |
| Awake SBP, mmHg | 125.5±12.2 | 126.5±13.3 | 0.237 |
| Awake DBP, mmHg | 84.5±9.6 | 85.2±10.4 | 0.303 |
| Sleep SBP, mmHg | 113.9±12.7 | 114.1±12.9 | 0.790 |
| Sleep DBP, mmHg | 74.2±9.6 | 75.2±10.3 | 0.168 |
| ACEI/ARB, n(%) | 187 (41.0%) | 190 (44.6%) | 0.281 |
| Beta-blocker, n(%) | 188 (41.2%) | 210 (49.3%) | 0.016 |
| CCB, n(%) | 186 (40.8%) | 183 (43.0%) | 0.514 |
| Thiazide, n(%) | 73 (16.0%) | 70 (16.4%) | 0.865 |
| eGFR, mL/min/1.73m^2^ | 103.5±21.5 | 100.3±20.0 | 0.020 |
| Creatinine, mg/dL | 0.8±0.2 | 0.9±0.2 | 0.364 |
| Sodium, mmol/L | 141.2±2.8 | 141.3±2.5 | 0.684 |
| Potassium, mmol/L | 4.0±0.3 | 4.1±0.3 | 0.510 |
| Chloride, mmol/L | 102.2±2.8 | 102.1±2.5 | 0.918 |
| Uric acid, mg/dL | 6.7±1.7 | 6.6±1.8 | 0.446 |
| Total urine volume, mL | 1929.3±777.9 | 1884.7±735.8 | 0.383 |
| Urine creatinine, mg/day | 1556.6±495.0 | 1543.5±504.0 | 0.696 |
| Urine sodium, mmol/day | 174.2±68.0 | 163.0±73.9 | 0.020 |
| Urine potassium, mmol/day | 46.1±17.2 | 46.2±17.3 | 0.905 |
| Urine chloride, mmol/day | 154.3±61.0 | 145.7±69.6 | 0.053 |

ACEI, angiotensin converting enzyme inhibitor; ARB, angiotensin receptor blocker; BMI, body mass index; CCB, calcium channel blocker; DBP, diastolic blood pressure; eGFR, estimated glomerular filtration rate; SBP, systolic blood pressure.

**Supplementary Table 5.** Baseline characteristics according to *SLC12A3* rs7187932 genotype (cohort 1)

| **rs7187932** | **A/A (n=63)** | **A/G (n=307)** | **G/G (n=512)** | ***P*-value** |
| --- | --- | --- | --- | --- |
| Age, years | 39.2±7.9 | 40.5±7.5 | 41.5±6.9 | 0.024 |
| Male, n(%) | 46 (73.0%) | 212 (69.1%) | 351 (68.6%) | 0.770 |
| BMI, kg/m^2^ | 26.5±3.7 | 26.5±3.6 | 26.5±3.4 | 0.998 |
| Office SBP, mmHg | 130.6±13.5 | 129.5±13.9 | 128.6±15.2 | 0.447 |
| Office DBP mmHg | 88.4±9.9 | 85.8±11.4 | 86.2±11.9 | 0.251 |
| 24-hour SBP, mmHg | 127.9±16.1 | 124.8±14.5 | 126.6±14.3 | 0.129 |
| 24-hour DBP, mmHg | 85.3±12.3 | 84.1±11.7 | 85.3±11.8 | 0.383 |
| Awake SBP, mmHg | 127.5±11.6 | 125.5±12.4 | 126.1±13.1 | 0.475 |
| Awake DBP, mmHg | 85.7±9.4 | 84.5±9.8 | 85.0±10.2 | 0.652 |
| Sleep SBP, mmHg | 115.7±10.8 | 114.0±13.0 | 113.8±12.9 | 0.554 |
| Sleep DBP, mmHg | 75.5±8.8 | 74.3±10.0 | 74.8±10.1 | 0.628 |
| ACEI/ARB, n(%) | 23 (36.5%) | 125 (40.7%) | 229 (44.7%) | 0.311 |
| Beta-blocker, n(%) | 30 (47.6%) | 117 (38.1%) | 251 (49.0%) | 0.009 |
| CCB, n(%) | 27 (42.9%) | 119 (38.8%) | 223 (43.6%) | 0.398 |
| Thiazide, n(%) | 12 (19.0%) | 44 (14.3%) | 87 (17.0%) | 0.496 |
| eGFR, mL/min/1.73m^2^ | 105.4±19.5 | 103.7±21.7 | 100.5±20.4 | 0.040 |
| Creatinine, mg/dL | 0.8±0.2 | 0.8±0.2 | 0.9±0.2 | 0.362 |
| Sodium, mmol/L | 141.3±2.1 | 141.2±3.1 | 141.3±2.4 | 0.712 |
| Potassium, mmol/L | 4.1±0.2 | 4.1±0.3 | 4.0±0.3 | 0.913 |
| Chloride, mmol/L | 102.3±2.4 | 102.1±3.0 | 102.2±2.4 | 0.711 |
| Uric acid, mg/dL | 6.7±1.8 | 6.7±1.6 | 6.6±1.8 | 0.723 |
| Total urine volume, mL | 1951.4±833.1 | 1913.7±744.7 | 1898.8±757.2 | 0.861 |
| Urine creatinine, mg/day | 1644.7±575.7 | 1519.2±483.5 | 1557.3±497.6 | 0.170 |
| Urine sodium, mmol/day | 177.0±64.6 | 175.4±67.9 | 163.9±73.5 | 0.051 |
| Urine potassium, mmol/day | 47.9±15.3 | 45.9±16.6 | 46.1±17.9 | 0.700 |
| Urine chloride, mmol/day | 158.6±58.4 | 155.0±62.0 | 146.1±68.0 | 0.096 |

ACEI, angiotensin converting enzyme inhibitor; ARB, angiotensin receptor blocker; BMI, body mass index; CCB, calcium channel blocker; DBP, diastolic blood pressure; eGFR, estimated glomerular filtration rate; SBP, systolic blood pressure.

**Supplementary Table 6.** Baseline characteristics according to *SLC12A3* rs7187932 genotype (cohort 1)

| **rs7187932** | **A/A+A/G (n=370)** | **G/G (n=512)** | ***P*-value** |
| --- | --- | --- | --- |
| Age, years | 40.3±7.6 | 41.5±6.9 | 0.017 |
| Male, n(%) | 112 (30.3%) | 161 (31.4%) | 0.710 |
| BMI, kg/m^2^ | 26.5±3.6 | 26.5±3.4 | 0.994 |
| Office SBP, mmHg | 129.7±13.8 | 128.6±15.2 | 0.244 |
| Office DBP mmHg | 86.2±11.2 | 86.2±11.9 | 0.989 |
| 24-hour SBP, mmHg | 125.3±14.8 | 126.6±14.3 | 0.190 |
| 24-hour DBP, mmHg | 84.3±11.8 | 85.3±11.8 | 0.238 |
| Awake SBP, mmHg | 125.8±12.3 | 126.1±13.1 | 0.738 |
| Awake DBP, mmHg | 84.7±9.7 | 85.0±10.2 | 0.714 |
| Sleep SBP, mmHg | 114.3±12.6 | 113.8±12.9 | 0.552 |
| Sleep DBP, mmHg | 74.5±9.8 | 74.8±10.1 | 0.660 |
| ACEI/ARB, n(%) | 148 (40.0%) | 229 (44.7%) | 0.161 |
| Beta-blocker, n(%) | 147 (39.7%) | 251 (49.0%) | 0.006 |
| CCB, n(%) | 146 (39.5%) | 223 (43.6%) | 0.224 |
| Thiazide, n(%) | 56 (15.1%) | 87 (17.0%) | 0.460 |
| eGFR, mL/min/1.73m^2^ | 104.0±21.3 | 100.5±20.4 | 0.014 |
| Creatinine, mg/dL | 0.8±0.2 | 0.9±0.2 | 0.150 |
| Sodium, mmol/L | 141.2±3.0 | 141.3±2.4 | 0.522 |
| Potassium, mmol/L | 4.1±0.3 | 4.0±0.3 | 0.745 |
| Chloride, mmol/L | 102.1±2.9 | 102.2±2.4 | 0.630 |
| Uric acid, mg/dL | 6.7±1.7 | 6.6±1.8 | 0.418 |
| Total urine volume, mL | 1920.1±759.4 | 1898.8±757.2 | 0.681 |
| Urine creatinine, mg/day | 1540.6±501.8 | 1557.3±497.6 | 0.625 |
| Urine sodium, mmol/day | 175.7±67.2 | 163.9±73.5 | 0.013 |
| Urine potassium, mmol/day | 46.2±16.4 | 46.1±17.9 | 0.921 |
| Urine chloride, mmol/day | 155.6±61.3 | 146.1±68.0 | 0.031 |

ACEI, angiotensin converting enzyme inhibitor; ARB, angiotensin receptor blocker; BMI, body mass index; CCB, calcium channel blocker; DBP, diastolic blood pressure; eGFR, estimated glomerular filtration rate; SBP, systolic blood pressure.

**Supplementary Table 7.** Baseline characteristics according to *SLC12A3* rs16963397 genotype (cohort 2)

| **rs16963397** | **C/C (n=10)** | **C/G (n=33)** | **G/G (n=47)** | ***P*-value** |
| --- | --- | --- | --- | --- |
| Age, years | 46.4±9.5 | 54.9±10.3 | 54.4±14.7 | 0.163 |
| Male, n(%) | 5 (50.0%) | 9 (27.3%) | 31 (66.0%) | 0.003 |
| BMI, kg/m^2^ | 24.7±4.1 | 25.9±3.9 | 25.9±3.8 | 0.615 |
| SBP, mmHg | 137.5±16.7 | 145.5±16.6 | 143.6±17.8 | 0.442 |
| DBP, mmHg | 93.3±13.5 | 93.1±13.0 | 92.8±11.8 | 0.993 |
| eGFR, mL/min/1.73m^2^ | 118.8±27.6 | 107.9±26.1 | 103.3±19.4 | 0.149 |
| Creatinine, mg/dL | 0.7±0.1 | 0.7±0.2 | 0.8±0.2 | 0.022 |
| Sodium, mmol/L | 142.0±3.8 | 140.8±2.2 | 141.9±1.9 | 0.215 |
| Potassium, mmol/L | 3.6±0.3 | 3.8±0.3 | 3.8±0.4 | 0.489 |
| Uric acid, mg/dL | 5.4±2.3 | 5.4±1.2 | 5.8±1.1 | 0.415 |
| Glucose, mg/dL | 99.5±12.2 | 100.0±15.2 | 96.3±9.1 | 0.371 |

BMI, body mass index; DBP, diastolic blood pressure; SBP, systolic blood pressure.

**Supplementary Table 8.** Baseline characteristics according to *SLC12A3* rs16963397 genotype (cohort 2)

| **rs16963397** | **C/C+C/G (n=43)** | **G/G (n=47)** | ***P*-value** |
| --- | --- | --- | --- |
| Age, years | 52.9±10.6 | 54.4±14.7 | 0.584 |
| Male, n(%) | 14 (32.6%) | 31 (66.0%) | 0.002 |
| BMI, kg/m^2^ | 25.6±3.9 | 25.9±3.8 | 0.691 |
| SBP, mmHg | 143.6±16.8 | 143.6±17.8 | 0.999 |
| DBP, mmHg | 93.1±13.0 | 92.8±11.8 | 0.910 |
| eGFR, mL/min/1.73m^2^ | 110.4±26.6 | 103.3±19.4 | 0.152 |
| Creatinine, mg/dL | 0.7±0.2 | 0.8±0.2 | 0.005 |
| Sodium, mmol/L | 141.2±2.7 | 141.9±1.9 | 0.234 |
| Potassium, mmol/L | 3.7±0.3 | 3.8±0.4 | 0.647 |
| Uric acid, mg/dL | 5.4±1.6 | 5.8±1.1 | 0.183 |
| Glucose, mg/dL | 99.9±14.4 | 96.3±9.1 | 0.160 |

BMI, body mass index; DBP, diastolic blood pressure; SBP, systolic blood pressure.

**Supplementary Table 9.** Baseline characteristics according to *SLC12A3* rs13334864 genotype (cohort 2)

| **rs13334864** | **C/C (n=13)** | **C/T (n=32)** | **T/T (n=45)** | ***P*-value** |
| --- | --- | --- | --- | --- |
| Age, years | 47.5±9.3 | 54.9±10.2 | 54.6±15.0 | 0.169 |
| Male, n(%) | 6 (46.2%) | 10 (31.3%) | 29 (64.4%) | 0.016 |
| BMI, kg/m^2^ | 24.8±3.8 | 26.0±4.0 | 25.9±3.8 | 0.647 |
| SBP, mmHg | 138.8±15.9 | 144.7±17.3 | 144.1±17.6 | 0.586 |
| DBP, mmHg | 93.5±12.9 | 92.7±13.1 | 93.0±11.9 | 0.984 |
| eGFR, mL/min/1.73m^2^ | 122.6±30.6 | 105.2±23.0 | 103.2±19.4 | 0.025 |
| Creatinine, mg/dL | 0.7±0.2 | 0.7±0.2 | 0.8±0.2 | 0.033 |
| Sodium, mmol/L | 141.7±3.3 | 141.0±2.4 | 141.8±1.9 | 0.444 |
| Potassium, mmol/L | 3.7±0.3 | 3.8±0.3 | 3.7±0.3 | 0.500 |
| Uric acid, mg/dL | 5.3±2.2 | 5.5±1.1 | 5.8±1.1 | 0.434 |
| Glucose, mg/dL | 98.8±11.7 | 99.8±15.3 | 96.5±9.3 | 0.491 |

BMI, body mass index; DBP, diastolic blood pressure; SBP, systolic blood pressure.

**Supplementary Table 10.** Baseline characteristics according to *SLC12A3* rs13334864 genotype (cohort 2)

| **rs13334864** | **C/C+C/T (n=32)** | **T/T (n=45)** | ***P*-value** |
| --- | --- | --- | --- |
| Age, years | 52.8±10.4 | 54.6±15.0 | 0.494 |
| Male, n(%) | 16 (35.6%) | 29 (64.4%) | 0.006 |
| BMI, kg/m^2^ | 25.7±3.9 | 25.9±3.8 | 0.832 |
| SBP, mmHg | 143.0±16.9 | 144.1±17.6 | 0.770 |
| DBP, mmHg | 93.0±12.9 | 93.0±11.9 | 0.979 |
| eGFR, mL/min/1.73m^2^ | 110.3±26.3 | 103.2±19.4 | 0.150 |
| Creatinine, mg/dL | 0.7±0.2 | 0.8±0.2 | 0.012 |
| Sodium, mmol/L | 141.2±2.7 | 141.8±1.9 | 0.312 |
| Potassium, mmol/L | 3.8±0.3 | 3.7±0.3 | 0.788 |
| Uric acid, mg/dL | 5.4±1.5 | 5.8±1.1 | 0.236 |
| Glucose, mg/dL | 99.5±14.2 | 96.5±9.3 | 0.241 |

BMI, body mass index; DBP, diastolic blood pressure; SBP, systolic blood pressure.

**Supplementary Table 11.** Baseline characteristics according to *SLC12A3* rs7187932 genotype (cohort 2)

| **rs7187932** | **A/A (n=12)** | **A/G (n=32)** | **G/G (n=47)** | ***P*-value** |
| --- | --- | --- | --- | --- |
| Age, years | 47.4±9.8 | 54.2±10.2 | 54.9±14.8 | 0.189 |
| Male, n(%) | 5 (41.7%) | 11 (35.5%) | 29 (61.7%) | 0.063 |
| BMI, kg/m^2^ | 25.0±3.9 | 26.0±3.9 | 25.8±3.8 | 0.739 |
| SBP, mmHg | 140.5±15.7 | 143.1±16.8 | 144.7±18.0 | 0.754 |
| DBP, mmHg | 93.7±13.5 | 93.1±13.1 | 92.7±11.7 | 0.970 |
| eGFR, mL/min/1.73m^2^ | 124.4±31.2 | 107.0±21.7 | 102.0±20.0 | 0.010 |
| Creatinine, mg/dL | 0.7±0.2 | 0.7±0.2 | 0.8±0.2 | 0.014 |
| Sodium, mmol/L | 141.7±3.5 | 141.0 ±2.4 | 141.8±1.9 | 0.451 |
| Potassium, mmol/L | 3.7±0.3 | 3.8±0.3 | 3.7±0.3 | 0.610 |
| Uric acid, mg/dL | 5.1±2.1 | 5.7±1.3 | 5.8±1.1 | 0.284 |
| Glucose, mg/dL | 99.0±12.2 | 100.3±15.4 | 96.3±9.1 | 0.347 |

BMI, body mass index; DBP, diastolic blood pressure; SBP, systolic blood pressure.

**Supplementary Table 12.** Baseline characteristics according to *SLC12A3* rs7187932 genotype (cohort 2)

| **rs7187932** | **A/A+A/G (n=43)** | **G/G (n=47)** | ***P*-value** |
| --- | --- | --- | --- |
| Age, years | 52.3±10.4 | 54.9±14.8 | 0.339 |
| Male, n(%) | 16 (37.2%) | 29 (61.7%) | 0.020 |
| BMI, kg/m^2^ | 25.7±3.9 | 25.8±3.8 | 0.906 |
| SBP, mmHg | 142.4±16.3 | 144.7±18.0 | 0.536 |
| DBP, mmHg | 93.3±13.0 | 92.7±11.7 | 0.843 |
| eGFR, mL/min/1.73m^2^ | 111.9±25.6 | 102.0±20.0 | 0.045 |
| Creatinine, mg/dL | 0.7±0.2 | 0.8±0.2 | 0.005 |
| Sodium, mmol/L | 141.2±2.8 | 141.8±1.9 | 0.344 |
| Potassium, mmol/L | 3.8±0.3 | 3.7±0.3 | 0.661 |
| Uric acid, mg/dL | 5.5±1.6 | 5.8±1.1 | 0.344 |
| Glucose, mg/dL | 99.9±14.4 | 96.3±9.1 | 0.154 |

BMI, body mass index; DBP, diastolic blood pressure; SBP, systolic blood pressure.

**Supplementary Table 13.** Baseline characteristics according to *SLC12A3* rs16963397 genotype (cohort 3)

| **rs16963397** | **C/C (n=11)** | **C/G (n=63)** | **G/G (n=92)** | ***P*-value** |
| --- | --- | --- | --- | --- |
| Age, years | 74.5±11.8 | 69.0±11.8 | 67.4±13.6 | 0.205 |
| Male, n(%) | 8 (72.7%) | 42 (66.7%) | 49 (53.3%) | 0.163 |
| BMI, kg/m^2^ | 24.4±2.8 | 25.9±3.8 | 26.1±3.9 | 0.386 |
| Office SBP, mmHg | 135.8±15.4 | 131.0±18.0 | 129.6±17.1 | 0.509 |
| Office DBP, mmHg | 82.8±9.8 | 79.9±12.0 | 79.8±10.6 | 0.692 |
| 24-hour SBP, mmHg | 120.1±9.7 | 124.6±13.6 | 124.0±11.9 | 0.644 |
| 24-hour DBP, mmHg | 71.4±6.5 | 73.0±8.3 | 73.5±7.7 | 0.745 |
| Daytime SBP, mmHg | 123.1±9.4 | 126.1±13.2 | 125.9±12.5 | 0.821 |
| Daytime DBP, mmHg | 74.0±6.7 | 74.4±8.3 | 75.2±7.8 | 0.835 |
| Nighttime SBP, mmHg | 115.8±15.4 | 121.1±16.1 | 120.0±12.7 | 0.613 |
| Nighttime DBP, mmHg | 66.6±9.9 | 70.2±9.9 | 69.8±9.3 | 0.620 |
| ACEI/ARB, n(%) | 7 (63.6%) | 39 (61.9%) | 56 (60.9%) | 0.980 |
| Beta-blocker, n(%) | 2 (18.2%) | 15 (23.8%) | 27 (29.3%) | 0.604 |
| CCB, n(%) | 10 (90.9%) | 44 (69.8%) | 61 (66.3%) | 0.245 |
| Thiazide, n(%) | 7 (63.6%) | 22 (34.9%) | 28 (30.4%) | 0.090 |
| eGFR, mL/min/1.73m² | 85.0±21.3 | 85.4±22.0 | 83.2±22.6 | 0.818 |
| Creatinine, mg/dL | 0.9±0.2 | 0.9±0.2 | 0.9±0.2 | 0.967 |
| Sodium, mmol/L | 140.7±1.8 | 141.8±2.6 | 141.7±2.8 | 0.447 |
| Potassium, mmol/L | 3.7±0.4 | 3.7±0.4 | 3.7±0.5 | 0.996 |
| Uric acid, mg/dL | 6.6±2.1 | 6.6±1.5 | 6.2±1.5 | 0.341 |
| Glucose, mg/dL | 102.3±8.4 | 99.7±9.9 | 99.6±10.4 | 0.708 |

ACEI, angiotensin converting enzyme inhibitor; ARB, angiotensin receptor blocker; BMI, body mass index; CCB, calcium channel blocker; DBP, diastolic blood pressure; eGFR, estimated glomerular filtration rate; SBP, systolic blood pressure.

**Supplementary Table 14.** Baseline characteristics according to *SLC12A3* rs16963397 genotype (cohort 3)

| **rs16963397** | **C/C+C/G (n=74)** | **G/G (n=92)** | ***P*-value** |  | **C/C (n=11)** | **C/G+G/G (n=155)** | ***P*-value** |
| --- | --- | --- | --- | --- | --- | --- | --- |
| Age, years | 69.8±11.9 | 67.4±13.6 | 0.223 |  | 74.5±11.8 | 68.1±12.9 | 0.104 |
| Male, n(%) | 50 (67.6%) | 49 (53.3%) | 0.062 |  | 8 (72.7%) | 91 (58.7%) | 0.280 |
| BMI, kg/m^2^ | 25.7±3.7 | 26.1±3.9 | 0.478 |  | 24.4±2.8 | 26.0±3.9 | 0.096 |
| Office SBP, mmHg | 131.7±17.6 | 129.6±17.1 | 0.428 |  | 135.8±15.4 | 130.1±17.5 | 0.264 |
| Office DBP, mmHg | 80.3±11.7 | 79.8±10.6 | 0.773 |  | 82.8±9.8 | 79.8±11.2 | 0.352 |
| 24-hour SBP, mmHg | 124.0±13.1 | 124.0±11.9 | 0.988 |  | 120.1±9.7 | 124.2±12.6 | 0.286 |
| 24-hour DBP, mmHg | 72.8±8.1 | 73.5±7.7 | 0.588 |  | 71.4±6.5 | 73.3±7.9 | 0.442 |
| Daytime SBP, mmHg | 125.7±12.7 | 125.9±12.5 | 0.903 |  | 123.1±9.4 | 126.0±12.7 | 0.438 |
| Daytime DBP, mmHg | 74.3±8.0 | 75.2±7.8 | 0.557 |  | 74.0±6.7 | 74.8±8.0 | 0.744 |
| Nighttime SBP, mmHg | 120.3±16.0 | 120.0±12.7 | 0.885 |  | 115.8±15.4 | 120.4±14.2 | 0.427 |
| Nighttime DBP, mmHg | 69.7±9.9 | 69.8±9.3 | 0.970 |  | 66.6±9.9 | 69.9±9.5 | 0.386 |
| ACEI/ARB, n(%) | 46 (62.2%) | 56 (60.9%) | 0.865 |  | 7 (63.6%) | 95 (61.3%) | 0.574 |
| Beta-blocker, n(%) | 17 (23.0%) | 27 (29.3%) | 0.355 |  | 2 (18.2%) | 42 (27.1%) | 0.404 |
| CCB, n(%) | 54 (73.0%) | 61 (66.3%) | 0.355 |  | 10 (90.9%) | 105 (67.7%) | 0.096 |
| Thiazide, n(%) | 29 (39.2%) | 28 (30.4%) | 0.238 |  | 7 (63.6%) | 50 (32.3%) | 0.040 |
| eGFR, mL/min/1.73m² | 85.4±21.8 | 83.2±22.6 | 0.525 |  | 85.0 ± 21.3 | 84.1±22.3 | 0.895 |
| Creatinine, mg/dL | 0.9±0.2 | 0.9±0.2 | 0.818 |  | 0.9 ± 0.2 | 0.9±0.2 | 0.860 |
| Sodium, mmol/L | 141.7±2.6 | 141.7±2.8 | 0.920 |  | 140.7 ± 1.8 | 141.8±2.7 | 0.105 |
| Potassium, mmol/L | 3.7±0.4 | 3.7±0.5 | 0.966 |  | 3.7 ± 0.4 | 3.7±0.4 | 0.923 |
| Uric acid, mg/dL | 6.6±1.6 | 6.2±1.5 | 0.144 |  | 6.6 ± 2.1 | 6.4±1.5 | 0.728 |
| Glucose, mg/dL | 100.1±9.7 | 99.6±10.4 | 0.757 |  | 102.3 ± 8.4 | 99.7±10.2 | 0.342 |

ACEI, angiotensin converting enzyme inhibitor; ARB, angiotensin receptor blocker; BMI, body mass index; CCB, calcium channel blocker; DBP, diastolic blood pressure; eGFR, estimated glomerular filtration rate; SBP, systolic blood pressure.

**Supplementary Table 15.** Baseline characteristics according to *SLC12A3* rs13334864 genotype (cohort 3)

| **rs13334864** | **C/C (n=11)** | **C/T (n=66)** | **T/T (n=89)** | ***P*-value** |
| --- | --- | --- | --- | --- |
| Age, years | 74.5±11.8 | 69.0±11.6 | 67.3±13.8 | 0.198 |
| Male, n(%) | 8 (72.7%) | 45 (68.2%) | 46 (51.7%) | 0.077 |
| BMI, kg/m^2^ | 24.4±2.8 | 25.9±3.8 | 26.1±4.0 | 0.397 |
| Office SBP, mmHg | 135.8±15.4 | 130.8±17.8 | 129.7±17.3 | 0.533 |
| Office DBP, mmHg | 82.8±9.8 | 80.1±11.9 | 79.7±10.7 | 0.673 |
| 24-hour SBP, mmHg | 120.1±9.7 | 124.4±13.4 | 124.1±11.9 | 0.660 |
| 24-hour DBP, mmHg | 71.4±6.5 | 73.1±8.2 | 73.5±7.7 | 0.759 |
| Daytime SBP, mmHg | 123.1±9.4 | 125.8±13.1 | 126.1±12.5 | 0.817 |
| Daytime DBP, mmHg | 74.0±6.7 | 74.5±8.2 | 75.1±7.9 | 0.877 |
| Nighttime SBP, mmHg | 115.8±15.4 | 121.0±16.0 | 120.0±12.7 | 0.623 |
| Nighttime DBP, mmHg | 66.6±9.9 | 70.3±9.7 | 69.7±9.4 | 0.610 |
| ACEI/ARB, n(%) | 7 (63.6%) | 42 (63.6%) | 53 (59.6%) | 0.865 |
| Beta-blocker, n(%) | 2 (18.2%) | 18 (27.3%) | 24 (27.0%) | 0.810 |
| CCB, n(%) | 10 (90.9%) | 45 (68.2%) | 60 (67.4%) | 0.272 |
| Thiazide, n(%) | 7 (63.6%) | 23 (34.8%) | 27 (30.3%) | 0.089 |
| eGFR, mL/min/1.73m² | 85.0±21.3 | 85.0±21.6 | 83.4±23.0 | 0.900 |
| Creatinine, mg/dL | 0.9±0.2 | 0.9±0.2 | 0.9±0.2 | 0.897 |
| Sodium, mmol/L | 140.7±1.8 | 141.6±3.2 | 141.9±2.3 | 0.362 |
| Potassium, mmol/L | 3.7±0.4 | 3.7±0.4 | 3.7±0.5 | 0.990 |
| Uric acid, mg/dL | 6.6±2.1 | 6.6±1.5 | 6.2±1.5 | 0.232 |
| Glucose, mg/dL | 102.3±8.4 | 100.3±10.5 | 99.1±10.0 | 0.546 |

ACEI, angiotensin converting enzyme inhibitor; ARB, angiotensin receptor blocker; BMI, body mass index; CCB, calcium channel blocker; DBP, diastolic blood pressure; eGFR, estimated glomerular filtration rate; SBP, systolic blood pressure.

**Supplementary Table 16.** Baseline characteristics according to *SLC12A3* rs13334864 genotype (cohort 3)

| **rs13334864** | **C/C+C/T (n=77)** | **T/T (n=89)** | ***P*-value** |  | **C/C (n=11)** | **C/T+T/T (n=155)** | ***P*-value** |
| --- | --- | --- | --- | --- | --- | --- | --- |
| Age, years | 69.8±11.7 | 67.3±13.8 | 0.215 |  | 74.5±11.8 | 68.1±12.9 | 0.104 |
| Male, n(%) | 53 (68.8%) | 46 (51.7%) | 0.025 |  | 8 (72.7%) | 91 (58.7%) | 0.280 |
| BMI, kg/m^2^ | 25.7±3.7 | 26.1±4.0 | 0.553 |  | 24.4±2.8 | 26.0±3.9 | 0.096 |
| Office SBP, mmHg | 131.5±17.5 | 129.7±17.3 | 0.491 |  | 135.8±15.4 | 130.1±17.5 | 0.264 |
| Office DBP, mmHg | 80.5±11.6 | 79.7±10.7 | 0.640 |  | 82.8±9.8 | 79.8±11.2 | 0.352 |
| 24-hour SBP, mmHg | 123.8±13.0 | 124.1±11.9 | 0.899 |  | 120.1±9.7 | 124.2±12.6 | 0.286 |
| 24-hour DBP, mmHg | 72.8±8.0 | 73.5±7.7 | 0.629 |  | 71.4±6.5 | 73.3±7.9 | 0.442 |
| Daytime SBP, mmHg | 125.5±12.6 | 126.1±12.5 | 0.776 |  | 123.1±9.4 | 126.0±12.7 | 0.438 |
| Daytime DBP, mmHg | 74.4±8.0 | 75.1±7.9 | 0.625 |  | 74.0±6.7 | 74.8±8.0 | 0.744 |
| Nighttime SBP, mmHg | 120.3±15.9 | 120.0±12.7 | 0.906 |  | 115.8±15.4 | 120.4±14.2 | 0.427 |
| Nighttime DBP, mmHg | 69.8±9.7 | 69.7±9.4 | 0.977 |  | 66.6±9.9 | 69.9±9.5 | 0.386 |
| ACEI/ARB, n(%) | 49 (63.6%) | 53 (59.6%) | 0.590 |  | 7 (63.6%) | 95 (61.3%) | 0.574 |
| Beta-blocker, n(%) | 20 (26.0%) | 24 (27.0%) | 0.885 |  | 2 (18.2%) | 42 (27.1%) | 0.404 |
| CCB, n(%) | 55 (71.4%) | 60 (67.4%) | 0.576 |  | 10 (90.9%) | 105 (67.7%) | 0.096 |
| Thiazide, n(%) | 30 (39.0%) | 27 (30.3%) | 0.243 |  | 7 (63.6%) | 50 (32.3%) | 0.040 |
| eGFR, mL/min/1.73m² | 85.0±21.4 | 83.4±23.0 | 0.644 |  | 85.0±21.3 | 84.1±22.3 | 0.895 |
| Creatinine, mg/dL | 0.9±0.2 | 0.9±0.2 | 0.641 |  | 0.9±0.2 | 0.9±0.2 | 0.860 |
| Sodium, mmol/L | 141.5±3.0 | 141.9±2.3 | 0.312 |  | 140.7±1.8 | 141.8±2.7 | 0.105 |
| Potassium, mmol/L | 3.7±0.4 | 3.7±0.5 | 0.935 |  | 3.7±0.4 | 3.7±0.4 | 0.923 |
| Uric acid, mg/dL | 6.6±1.6 | 6.2±1.5 | 0.087 |  | 6.6±2.1 | 6.4±1.5 | 0.728 |
| Glucose, mg/dL | 100.6±10.2 | 99.1±10.0 | 0.353 |  | 102.3±8.4 | 99.7±10.2 | 0.342 |

ACEI, angiotensin converting enzyme inhibitor; ARB, angiotensin receptor blocker; BMI, body mass index; CCB, calcium channel blocker; DBP, diastolic blood pressure; eGFR, estimated glomerular filtration rate; SBP, systolic blood pressure.

**Supplementary Table 17.** Baseline characteristics according to *SLC12A3* rs7187932 genotype (cohort 3)

| **rs7187932** | **A/A (n=7)** | **A/G (n=66)** | **G/G (n=93)** | ***P*-value** |
| --- | --- | --- | --- | --- |
| Age, years | 75.4±12.6 | 68.4±11.4 | 68.0±13.9 | 0.341 |
| Male, n(%) | 6 (85.7%) | 43 (65.2%) | 50 (53.8%) | 0.126 |
| BMI, kg/m^2^ | 24.5±2.9 | 25.8±3.8 | 26.1±3.9 | 0.584 |
| Office SBP, mmHg | 132.6±14.0 | 131.3±18.1 | 129.8±17.2 | 0.837 |
| Office DBP, mmHg | 79.4±9.5 | 80.5±12.1 | 79.7±10.6 | 0.900 |
| 24-hour SBP, mmHg | 119.3±11.0 | 124.8±13.1 | 123.7±12.1 | 0.575 |
| 24-hour DBP, mmHg | 71.2±7.4 | 73.3±8.1 | 73.3±7.7 | 0.812 |
| Daytime SBP, mmHg | 121.5±10.5 | 126.4±12.7 | 125.8±12.7 | 0.664 |
| Daytime DBP, mmHg | 73.2±7.7 | 74.8±8.2 | 74.9±7.8 | 0.873 |
| Nighttime SBP, mmHg | 117.3±16.2 | 121.3±15.8 | 119.5±12.9 | 0.696 |
| Nighttime DBP, mmHg | 67.5±10.4 | 70.4±9.7 | 69.4±9.4 | 0.722 |
| ACEI/ARB, n(%) | 5 (71.4%) | 41 (62.1%) | 56 (60.2%) | 0.833 |
| Beta-blocker, n(%) | 2 (28.6%) | 16 (24.2%) | 26 (28.0%) | 0.865 |
| CCB, n(%) | 6 (85.7%) | 46 (69.7%) | 63 (67.7%) | 0.607 |
| Thiazide, n(%) | 5 (71.4%) | 24 (36.4%) | 28 (30.1%) | 0.077 |
| eGFR, mL/min/1.73m² | 84.1±21.4 | 85.1±22.1 | 83.5±22.6 | 0.906 |
| Creatinine, mg/dL | 0.9±0.2 | 0.9±0.2 | 0.9±0.2 | 0.851 |
| Sodium, mmol/L | 140.4±1.4 | 141.6±3.2 | 141.9±2.3 | 0.345 |
| Potassium, mmol/L | 3.7±0.4 | 3.7±0.4 | 3.7±0.5 | 0.999 |
| Uric acid, mg/dL | 6.6±2.0 | 6.6±1.6 | 6.2±1.5 | 0.185 |
| Glucose, mg/dL | 102.7±9.0 | 100.5±10.7 | 99.2±9.8 | 0.535 |

ACEI, angiotensin converting enzyme inhibitor; ARB, angiotensin receptor blocker; BMI, body mass index; CCB, calcium channel blocker; DBP, diastolic blood pressure; eGFR, estimated glomerular filtration rate; SBP, systolic blood pressure.

**Supplementary Table 18.** Baseline characteristics according to *SLC12A3* rs7187932 genotype (cohort 3)

| **rs7187932** | **A/A+A/G (n=73)** | **G/G (n=93)** | ***P*-value** |  | **A/A (n=7)** | **A/G+G/G (n=159)** | ***P*-value** |
| --- | --- | --- | --- | --- | --- | --- | --- |
| Age, years | 69.1±11.6 | 68.0±13.9 | 0.588 |  | 75.4±12.6 | 68.2±12.9 | 0.184 |
| Male, n(%) | 49 (67.1%) | 50 (53.8%) | 0.082 |  | 6 (85.7%) | 93 (58.5%) | 0.148 |
| BMI, kg/m^2^ | 25.7±3.7 | 26.1±3.9 | 0.559 |  | 24.5±2.9 | 26.0±3.9 | 0.238 |
| Office SBP, mmHg | 131.4±17.6 | 129.8±17.2 | 0.572 |  | 132.6±14.0 | 130.4±17.5 | 0.706 |
| Office DBP, mmHg | 80.4±11.8 | 79.7±10.6 | 0.700 |  | 79.4±9.5 | 80.1±11.2 | 0.870 |
| 24-hour SBP, mmHg | 124.3±12.9 | 123.7±12.1 | 0.812 |  | 119.3±11.0 | 124.2±12.5 | 0.334 |
| 24-hour DBP, mmHg | 73.1±8.0 | 73.3±7.7 | 0.854 |  | 71.2±7.4 | 73.3±7.9 | 0.521 |
| Daytime SBP, mmHg | 125.9±12.5 | 125.8±12.7 | 0.951 |  | 121.5±10.5 | 126.0±12.6 | 0.349 |
| Daytime DBP, mmHg | 74.6±8.1 | 74.9±7.8 | 0.817 |  | 73.2±7.7 | 74.9±7.9 | 0.619 |
| Nighttime SBP, mmHg | 120.9±15.7 | 119.5±12.9 | 0.587 |  | 117.3±16.2 | 120.3±14.2 | 0.679 |
| Nighttime DBP, mmHg | 70.1±9.7 | 69.4±9.4 | 0.692 |  | 67.5±10.4 | 69.8±9.5 | 0.611 |
| ACEI/ARB, n(%) | 46 (63.0%) | 56 (60.2%) | 0.713 |  | 5 (71.4%) | 97 (61.0%) | 0.450 |
| Beta-blocker, n(%) | 18 (24.7%) | 26 (28.0%) | 0.633 |  | 2 (28.6%) | 42 (26.4%) | 0.596 |
| CCB, n(%) | 52 (71.2%) | 63 (67.7%) | 0.628 |  | 6 (85.7%) | 109 (68.6%) | 0.309 |
| Thiazide, n(%) | 29 (39.7%) | 28 (30.1%) | 0.195 |  | 5 (71.4%) | 52 (32.7%) | 0.048 |
| eGFR, mL/min/1.73m² | 85.0±21.9 | 83.5±22.6 | 0.665 |  | 84.1±21.4 | 84.2±22.3 | 0.999 |
| Creatinine, mg/dL | 0.9±0.2 | 0.9±0.2 | 0.658 |  | 0.9±0.2 | 0.9±0.2 | 0.667 |
| Sodium, mmol/L | 141.5±3.1 | 141.9±2.3 | 0.340 |  | 140.4±1.4 | 141.8±2.7 | 0.048 |
| Potassium, mmol/L | 3.7±0.4 | 3.7±0.5 | 0.972 |  | 3.7±0.4 | 3.7±0.4 | 0.969 |
| Uric acid, mg/dL | 6.6±1.6 | 6.2±1.5 | 0.067 |  | 6.6±2.0 | 6.4±1.6 | 0.732 |
| Glucose, mg/dL | 100.7±10.5 | 99.2±9.8 | 0.336 |  | 102.7±9.0 | 99.7±10.1 | 0.417 |

ACEI, angiotensin converting enzyme inhibitor; ARB, angiotensin receptor blocker; BMI, body mass index; CCB, calcium channel blocker; DBP, diastolic blood pressure; eGFR, estimated glomerular filtration rate; SBP, systolic blood pressure.
